# Seasonal and regional structuring of rhizosphere fungal communities in *Macadamia integrifolia*

**DOI:** 10.3389/fmicb.2025.1634222

**Published:** 2025-09-12

**Authors:** Ya Ning, Yuchun Chen, Zhonghua Wu, Tingmei Yang, Xiyong He, Hai Yue

**Affiliations:** ^1^Yunnan Institute of Tropical Crops, Xishuangbanna, China; ^2^Yunnan Macadamia Agricultural Engineering Research Center, Xishuangbanna, Yunnan, China

**Keywords:** *Macadamia integrifolia*, ITS sequencing, seasonal variation, cluster roots, microbial community structure, FUNGuild

## Abstract

**Introduction:**

Rhizosphere fungal communities are pivotal to plant nutrient acquisition, stress tolerance, and ecosystem functionality. However, the diversity and ecological roles of these communities in tropical cash crops like Macadamia integrifolia (macadamia) remain understudied—particularly how they respond to seasonal, geographic, and root-type variations. This knowledge gap hinders targeted management of rhizosphere microbes for sustainable macadamia production.

**Methods:**

To address this, we examined the spatiotemporal structuring of rhizosphere fungal communities in M. integrifolia across four major production regions in Yunnan Province, China (Changning, Yingjiang, Lancang, Yunxian). We accounted for three key variables: season (dry season: November–April; rainy season: May–October), root type (normal roots vs. cluster roots), and geography. A total of 80 soil samples were collected (4 regions × 2 seasons × 2 root types × 5 biological replicates). High-throughput sequencing of the fungal Internal Transcribed Spacer (ITS) region was used to analyze community composition, diversity, and functional guilds; co-occurrence network analysis and PERMANOVA were also employed to interpret community dynamics.

**Results:**

Season and geographic location significantly shaped fungal community structure, while the effect of root type was context-dependent. Fungal diversity was higher in the rainy season, with Ascomycota (55–65%), Basidiomycota (20–30%), and Mortierellomycota (5–10%) as the dominant phyla. Cluster roots enriched symbiotic and beneficial taxa: Glomus and Trichoderma were 1.8- and 2.3-fold more abundant in cluster roots than in normal roots, respectively. PERMANOVA confirmed significant effects of season and region on community structure (*p* = 0.001). Co-occurrence networks showed seasonal shifts in core taxa: dry-season networks were dominated by Talaromyces and Penicillium (Ascomycota), while rainy-season networks featured Cladosporium (Ascomycota) and Mortierellaceae (Mortierellomycota)—with 35% of edges being negative interactions in the rainy season, indicating heightened resource competition. FUNGuild predictions revealed saprotrophic fungi were predominant (50–55%), with a 10% higher proportion in rainy-season samples than in dry-season samples.

**Discussion:**

This study clarifies the dynamic and region-specific nature of M. integrifolia rhizosphere fungal communities, highlighting how environmental factors drive their composition and function. These findings fill a critical knowledge gap and provide a foundational framework for future research on rhizosphere fungi in macadamia cultivation, supporting efforts to improve crop sustainability.

## 1 Introduction

The root surfaces of plants and the soil in which they are embedded together constitute a complex environment, referred to as the rhizosphere. This environment is characterized by high levels of microbial activity, sustained by the release of nutrients through plant root exudates (Matilla et al., 2007). A wide array of organisms, encompassing fungi, bacteria, archaea, and diminutive soil-dwelling creatures (e.g., nematodes), inhabit the inter-root zone of plants. Previous studies have highlighted that rhizosphere microbial diversity (including bacteria, fungi, and archaea) is tightly linked to plant health, with bacteria often involved in nutrient cycling and fungi in symbiosis and organic matter decomposition. These organisms play a pivotal role in various aspects of plant development, including stress tolerance, nutrient acquisition, and soil health (Bandopadhyay et al., 2024; Berendsen et al., 2012; Pii et al., 2015). Bulk soil refers to soil not directly influenced by root exudates or root-derived signals, characterized by lower microbial activity and less intimate associations with plant roots compared to rhizosphere soil. In comparison with bulk soil, rhizosphere soils have been found to contain more diverse microbial communities that are closely associated with plants, particularly fungal taxa (Ling et al., 2022). These fungi perform a number of indispensable functions, including the decomposition of organic matter, the transformation of mineral nutrients, the formation of symbiotic associations with roots, and the suppression of pathogenic organisms (Nicolás et al., 2018; Xu et al., 2024; Shah et al., 2016). It has been demonstrated that root exudates, which include organic acids, sugars and signalling molecules (Zhang et al., 2014), can selectively recruit beneficial microbes, such as arbuscular mycorrhizal fungi (AMF) and biocontrol Trichoderma species (Wu et al., 2025; Zhou et al., 2020; Singh et al., 2024), while simultaneously inhibiting the colonization of pathogenic fungi. This process establishes a highly specific microbial barrier (Jin et al., 2024). The “plant–fungus” interaction network exerts a significant influence on the performance of individual plants and, moreover, plays a pivotal role in shaping the functionality of ecosystems by modulating the structure of the soil food web (Parisy et al., 2024).

Macadamia nuts are rich in unsaturated fatty acids, proteins, and various trace elements, and are known as the ‘king of nuts’ (Lin et al., 2022). Macadamia is a widely utilized ingredient in the food and nutraceutical industries, with a consistently rising demand in the market (Du et al., 2010). Currently, China is the leading and quickest growing nation for macadamia plantations globally (Shuai et al., 2021). However, the continuous expansion of the planting area has led to increased vulnerability to diseases, resulting in unstable yields and quality. This has imposed significant restrictions on the sustainable development of the macadamia nut industry (Yao et al., 2024). Traditional water and nutrient management practices have proven ineffective in overcoming the inherent limitations of root nutrient uptake efficiency and stress resistance, thereby imposing substantial constraints on the crop’s sustainable development (Gu et al., 2022). It is evident that rhizosphere fungi, in their capacity as pivotal mediators of plant nutrient acquisition and environmental adaptability (Lutz et al., 2023; Hestrin et al., 2019), may serve as a critical leverage point for surmounting the prevailing bottlenecks in yield and quality. The geographic characteristics of Yunnan Province create distinct geographical and climatic differences, most notably the seasonal contrast between the dry season (November to April) and the rainy season (May to October) (Lai et al., 2023). Dramatic fluctuations in temperature, precipitation and humidity occur during the rainy season. The natural environmental heterogeneity thus engendered provides an ideal framework for investigating how rhizosphere microorganisms respond to varying climatic conditions (Boyle et al., 2024). As demonstrated in previous studies, climatic factors and geographic location have been shown to exert a significant influence on the composition and function of soil microbial communities (Gong et al., 2024; Naylor et al., 2020). For *Macadamia integrifolia*, an introduced species, its rhizosphere fungal communities may exhibit distinct spatial distributions and adaptive strategies across different climatic and edaphic environments.

Despite the considerable growth in research on rhizosphere microorganisms and their interactions with plants, systematic studies on perennial economic tree species such as *Macadamia integrifolia* – particularly regarding their rhizosphere fungal communities – remain extremely limited. This dearth of research considerably hinders the practical manipulation and utilization of rhizosphere microbes in macadamia cultivation. Conventional wisdom held that macadamia, a member of the Proteaceae family, was not capable of forming typical cluster roots. However, extant research has demonstrated that macadamia is indeed capable of developing cluster roots, which exhibit distinctive morphological and functional characteristics (Zhao et al., 2021). It is noteworthy that the fungal communities present in the rhizosphere of these cluster roots differ considerably from those observed in normal roots (Gong et al., 2024). Moreover, the presence of AMF has been detected in the cluster root zones. These roots exhibit distinctive exudation profiles, which may facilitate the attraction of particular fungal taxa through root-secreted compounds. Despite these findings, no comprehensive studies have yet clarified the seasonal and spatial dynamics of fungal community differences between cluster and normal roots, nor has a quantitative framework for comparison been established. This knowledge gap hinders our ability to understand the ecological regulatory functions of the macadamia rhizosphere, thereby restricting the development of targeted microbial strategies for sustainable cultivation.

This study aims to investigate how seasonal changes (dry season and rainy season), geographical location, and root type (normal roots and cluster roots) influence the diversity, composition, and functional characteristics of the rhizosphere fungal community in macadamia nuts. Based on existing research gaps, we propose three hypotheses: (1) Seasonal factors (especially precipitation and temperature fluctuations) and geographical variables (elevation, microclimate) are the primary drivers of fungal community structure, and their interactions amplify these effects; (2) Cluster roots can selectively enrich specific symbiotic fungal groups through root exudates, but this enrichment is geographically dependent–it is more pronounced during the rainy season and in regions with richer soil nutrients; (3) Seasonal and regional changes in fungal communities will synchronously alter functional group composition, with increased humidity and nutrients during the rainy season promoting saprophytic fungal proliferation, while drought stress during the dry season enriches drought-tolerant functional groups. Given the limited research on fungi associated with macadamia rhizosphere, this study aims to fill this gap and provide a research foundation for future studies on macadamia rhizosphere fungi.

## 2 Materials and methods

### 2.1 Study area and sample collection design

**Figure 1:**
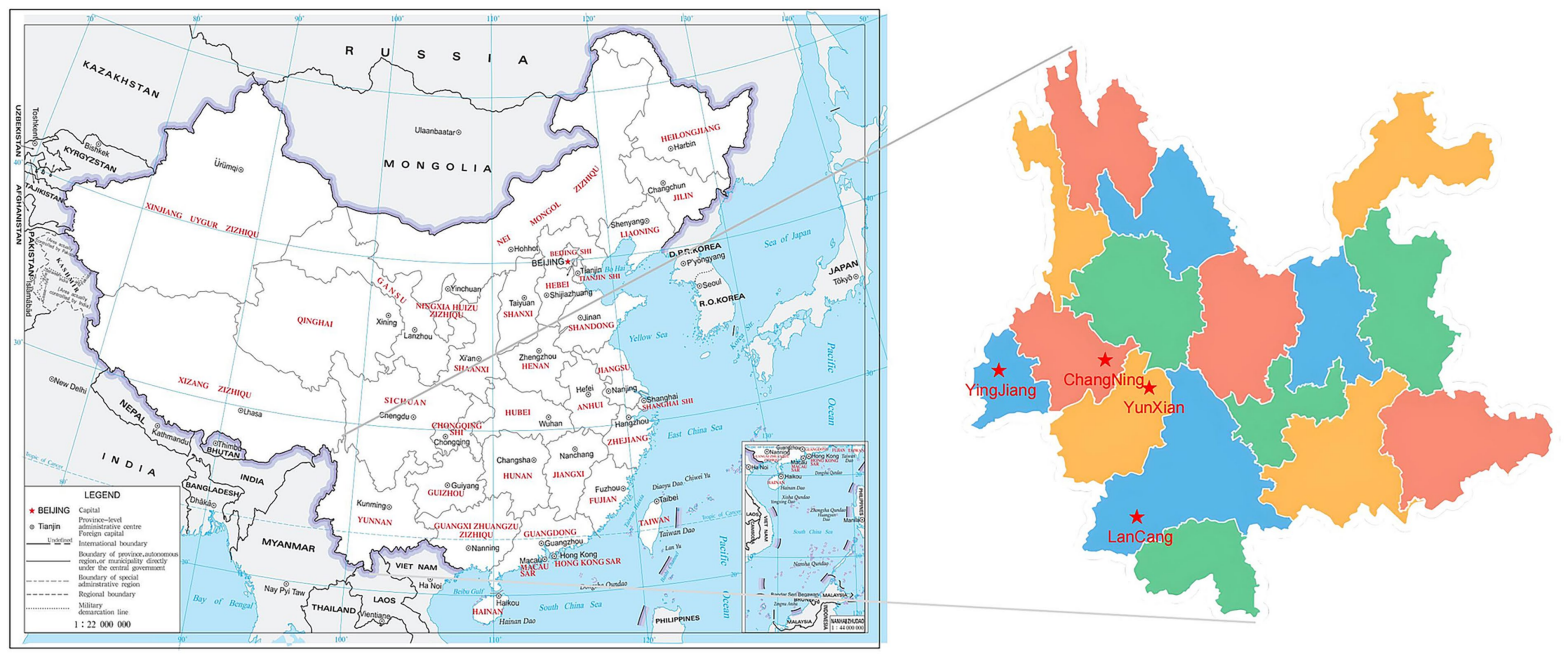
Geospatial distribution characteristics of the four sampling areas (Yingjiang, Changning, Lancang, and Yunxian, Yunnan Province) in this study.

In this study, rhizosphere soil samples were collected from both normal roots and cluster roots of *Macadamia integrifolia* across four major production regions in Yunnan Province–Changning (CN), Yingjiang (YJ), Lancang (LC), and Yunxian (YX)–during both the dry and rainy seasons. A total of 80 samples were obtained (4 regions × 2 seasons × 2 root types × 5 replicates), thereby establishing a multidimensional sampling framework incorporating seasonal, geographical, and root–type variation. These orchards exhibited distinct geographic and climatic characteristics (Figure 1; Table 1). As demonstrated in Table 1, each site exhibited significant seasonal fluctuations. For instance, in Changning, the air temperature increased from 10 to 17.4 °C in the dry season to 17.5–20.8 °C in the rainy season, while precipitation rose from 248 mm to 995 mm. Greater shifts were also observed in Yingjiang (14.2–21.8 °C and 186 mm vs. 22.5–24.4 °C and 1,366 mm), Lancang (13.2–21.8 °C and 195 mm vs. 23.5 °C and 1,429 mm), and Yunxian (13.8–24.4 °C and 273 mm vs. 26.9 °C and 1,094 mm). These seasonal contrasts were layered over clear spatial gradients. During the dry season, Changning exhibited the lowest temperatures, while Yunxian demonstrated the most extensive temperature range and the highest levels of precipitation. During the rainy season, Yunxian experienced the highest temperatures, while Lancang received the most precipitation, and Changning remained predominantly dry. The range of elevation was from 992 m in Lancang to 1,278 m in Yunxian. Collectively, these climatic and geographic gradients furnish a natural experimental framework for assessing how environmental variation influences the composition and dynamics of the macadamia rhizosphere mycobiome.

**Table 1:** Geographical location of the four Macadamia Nut Parks.

Location	Abbreviation	Location	Average elevation (m)	Dry season (November–April)		Rainy season (May–October)	
				Mean temperature (°C)	Precipitation (mm)	Mean temperature (°C)	Precipitation (mm)
ChangNing	CN	24°51′11″N 99°27′27″E	1,072	10–17.4	248	17.5–20.8	995
YingJiang	YJ	24°44′46″N 97°54′30″E	1,037	14.2–21.8	186	22.5–24.4	1,366
LanCang	LC	22°35′45″N 100°26′30″E	992	13.2–21.8	195	23.5	1,429
YunXian	YX	24°24′39″N 100°8′44″E	1,278	13.8–24.4	273	26.9	1,094

Sampling was conducted during two distinct seasons: the dry season (DS, November to April) and the rainy season (RS, May to October). In each orchard, sampling plots were meticulously selected based on flat terrain, uniform soil properties, and consistent cultivation and management practices. Each plot encompassed an area of 500 m × 500 m. All plots were exclusively planted with *Macadamia integrifolia* trees (cultivar O. C.) of similar age (≥8 years). At each site, rhizosphere soil was collected from two root types–normal roots (NR) and cluster roots (CR)–with five biological replicates per group, totaling 80 samples. Rhizosphere soil was collected by gently shaking roots to remove loose soil, then brushing adhering soil (2–5 mm from root surface) into sterile tubes. This follows standard protocols to ensure collection of root-influenced soil (Gong et al., 2024). The identification of root types was conducted *in situ* through the evaluation of tree morphological characteristics. Trees that were not exhibiting visible indications of disease were meticulously selected for the sampling process. A five-point method was employed to randomly collect soil tightly adhering (2–5 mm) to the root surface. Samples were promptly transferred to 50 mL sterile tubes, flash-frozen in liquid nitrogen, and stored at −80 °C for subsequent analysis. The implementation of sampling procedures was standardized across all regions.

### 2.2 DNA extraction and ITS amplicon sequencing

The procedures for DNA extraction and sequencing were carried out in accordance with previously established protocols (Gong et al., 2024). Briefly, genomic DNA was extracted from rhizosphere soil samples using the CTAB/SDS method, visualised on 1% agarose gels, and diluted to a final concentration of 1 ng/μL. The fungal internal transcribed spacer (ITS1–ITS2) region was amplified using the primers ITS1F (5′–CTTGGTCATTTAGAGGAAGTAA–3′) and ITS2R (5′–GCTGCGTTCTTCATCGATGC–3′) with Phusion® High–Fidelity PCR Master Mix under standard conditions. The presence of PCR products was confirmed on 2% agarose gels. Subsequently, the samples were combined in equimolar concentrations, and the purification process was executed using the Qiagen Gel Extraction Kit. The preparation of sequencing libraries was undertaken using the NEBNext® Ultra II DNA Library Prep Kit, and the quality of the libraries was subsequently assessed with a Qubit 2.0 Fluorometer and an Agilent Bioanalyzer 2,100. Paired–end sequencing (250 base pairs) was performed on the Illumina NovaSeq platform.

### 2.3 Sequencing data quality control and community diversity analysis

Paired–end reads were demultiplexed using unique sample barcodes and trimmed to remove barcode and primer sequences. Merged reads were generated using FLASH (v1.2.11), and quality filtering was performed with fastp (v0.20.0) to obtain high–quality tags. The detection and removal of chimeric sequences was conducted using Vsearch (v2.15.0) against the UNITE fungal database, yielding a set of effective tags. Amplicon sequence variant (ASV) inference and denoising were conducted using DADA2 within QIIME2 (v2020.6), followed by taxonomic and functional annotation. Subsequently, the ASV abundance tables were normalized to the sample with the lowest sequencing depth. Alpha diversity indices, including Observed_features, Chao1, Shannon, Simpson, Goods_coverage, and Pielou_e, were calculated in QIIME2 (Bolyen et al., 2019). Beta diversity was assessed using Bray–Curtis distances, and community composition was visualized via principal coordinate analysis (PCoA) and non–metric multidimensional scaling (NMDS). To assess the impact of season, region, and root type on fungal community structure, permutational multivariate analysis of variance (PERMANOVA) was conducted, leveraging the Adonis function with 999 permutations.

### 2.4 Analysis of community structure and prediction of fungal functions

The top ten most abundant fungal phyla and genera across all samples were used to generate stacked bar plots to visualize community composition trends. The identification of differentially abundant taxa was achieved through the implementation of Linear Discriminant Analysis Effect Size (LEfSe) with a threshold of LDA > 3.5 and *p* < 0.05, as well as through Random Forest modeling. The key genera were determined on the basis of their classification contribution and mean importance scores. The similarity percentage (SIMPER) analysis was employed to identify genera that contributed most to community dissimilarities across seasons and regions. The FUNGuild database was employed to perform functional prediction, thereby enabling the categorization of fungal taxa into ecological roles such as saprotrophic, pathogenic, or symbiotic. A comparative analysis was conducted, with the functional profiles (Modes) and ecological guilds (Guilds) being visualised.

### 2.5 Co–occurrence network construction and analysis

A co–occurrence network was constructed based on Spearman correlation coefficients (*ρ* > 0.6 and *p* < 0.01) among ASVs in order to identify key interactive taxa within the rhizosphere fungal community. The calculation of network topology metrics, incorporating node degree and betweenness centrality, was conducted using the igraph package in R (v1.2.6). Subsequently, the network was rendered visually using Gephi. Within the network, nodes are used to represent individual ASVs, with edge colors indicating the direction of correlation (red indicating positive and blue indicating negative).

### 2.6 Statistical analysis

The differences in alpha and beta diversity between groups were analyzed using the Kruskal–Wallis test in QIIME2. Furthermore, bioinformatic analysis was conducted using the OmicStudio tools, accessible at https://www.omicstudio.cn/tool (Lyu et al., 2023).

## 3 Results

### 3.1 Geographic environment of sampling sites and overview of sequencing data

After stringent quality control (Supplementary Table 1), an average of 79,799 high-quality reads per sample were retained (86.19% of raw reads, range: 62.31–96.39%). Q20 and Q30 scores exceeded 98.78 and 95.56%, respectively, with GC content (39.06–57.55%) consistent with fungal ITS regions. These metrics confirm the reliability of sequencing data for subsequent analyses.

### 3.2 Alpha and beta diversity of rhizosphere fungal communities

**Figure 2:**
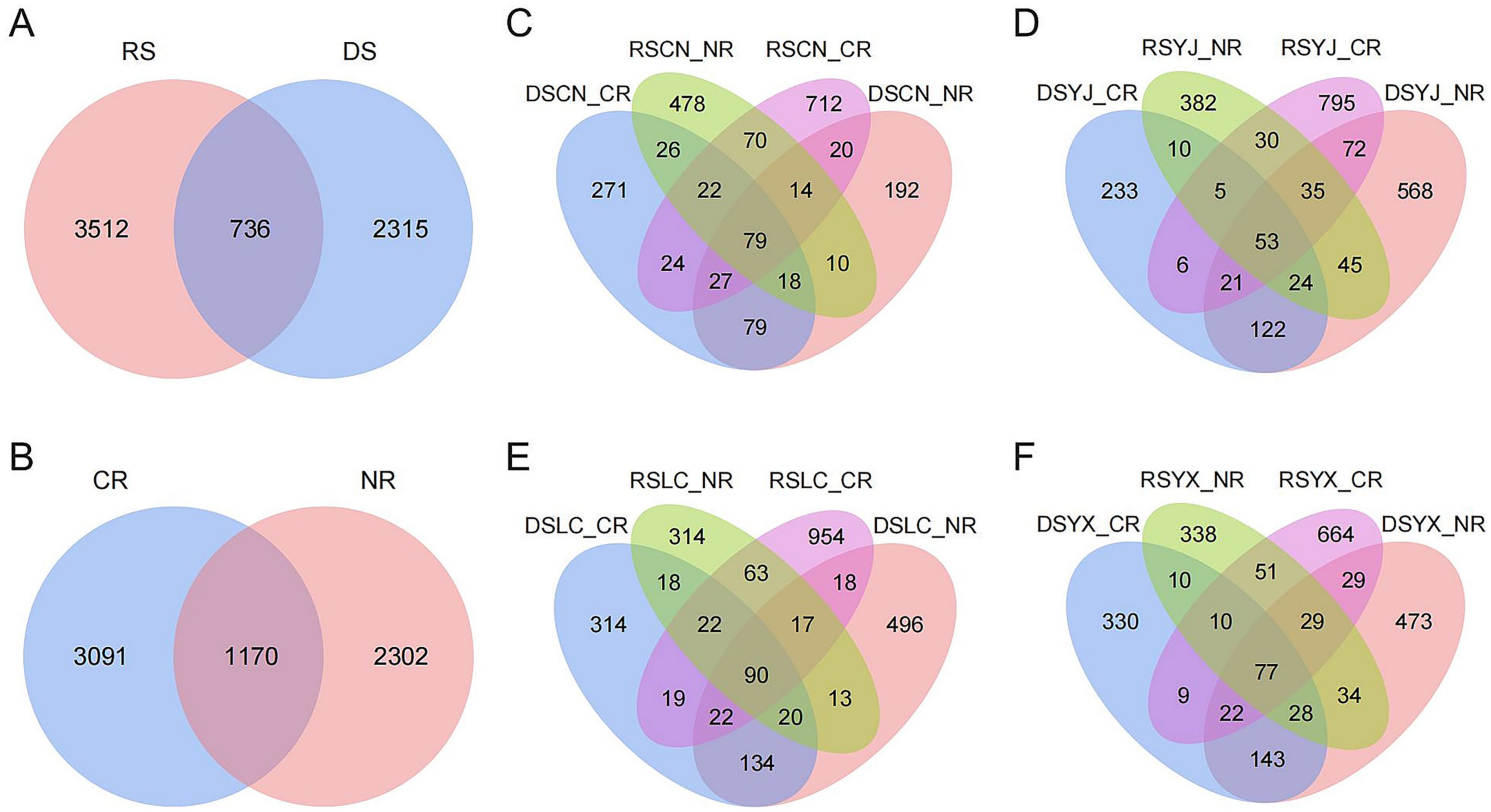
Wayne plots of rhizosphere fungal ASV distribution in macadamia nuts under different environments. **(A)** DS and RS; **(B)** CR and NR. Venn plots of ASV in different groups in four regions, Changning **(C)**, Yingjiang **(D)**, Lancang **(E)**, and Yunxian **(F)**. DS, dry season; RS, rainy season; CR, cluster roots; NR, normal roots. CN, Changning; YJ, Yingjiang; LC, Lancang; YX, Yunxian.

A total of 6,563 fungal ASVs were identified across all samples. Venn diagram analyses revealed substantial differences among environmental groups (Figure 2). A mere 11.21% of ASVs were shared between the DS and RS, while 35.27 and 53.51% were unique, respectively. In a similar manner, CR and NR samples exhibited a mere 17.83% of ASVs in common. Within each site, combinations of season and root type also exhibited high ASV specificity. The findings indicate that seasonality, root morphology, and site conditions interact to shape a highly specialized and context–dependent rhizosphere mycobiome.

**Figure 3:**
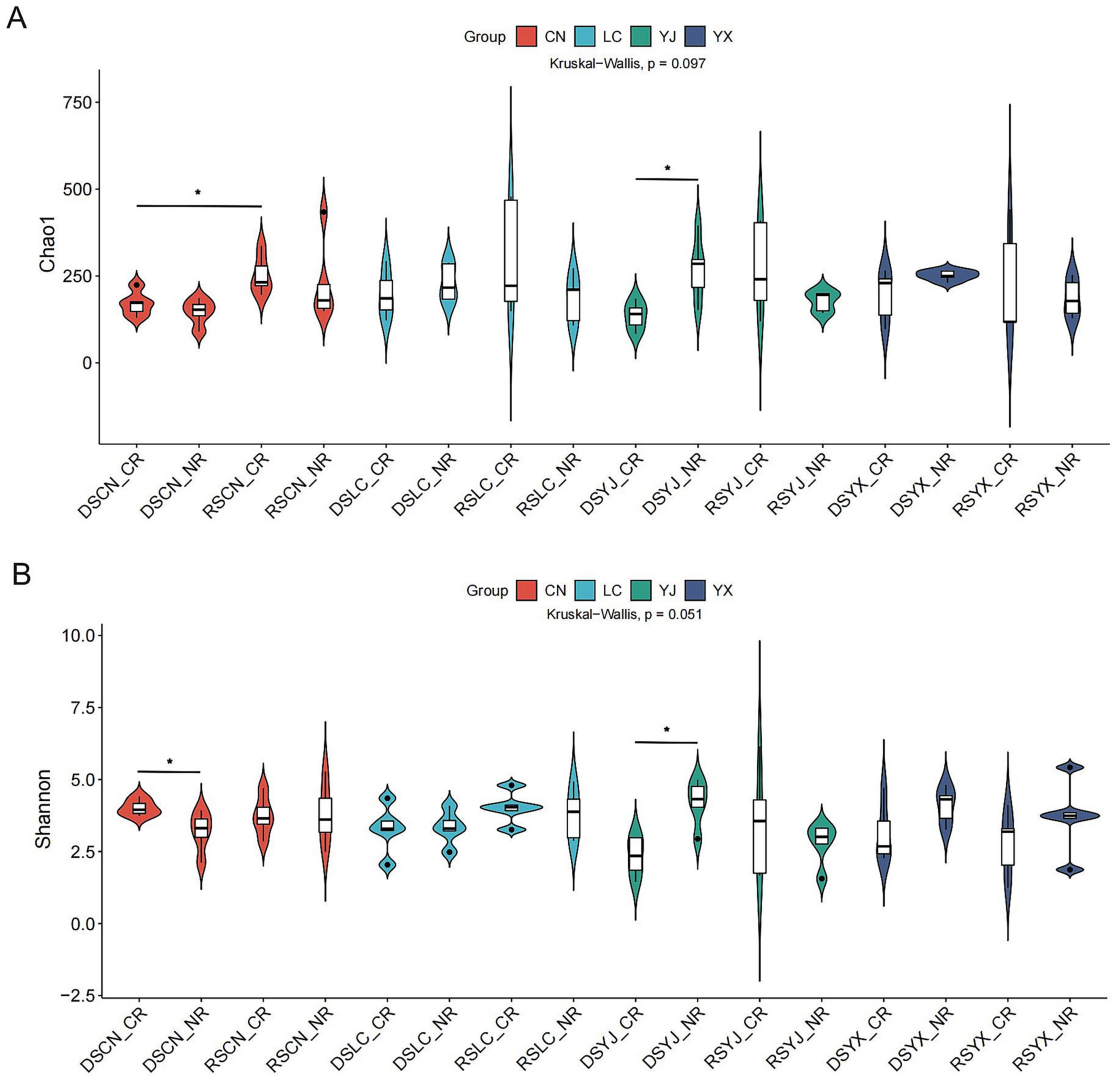
Analysis of alpha diversity of rhizosphere fungal communities of macadamia nuts in different environments. Violin plots of Chao1 **(A)** and Shannon **(B)** for different groups. DS, dry season; RS, rainy season; CR, cluster roots; NR, normal roots; CN, Changning; YJ, Yingjiang; LC, Lancang; YX, Yunxian. Differences were analyzed using the nonparametric test of KW. *Indicates significant difference at *p* < 0.05.

Alpha diversity varied across regions, seasons, and root types (Figures 3A,B), with 6,563 fungal ASVs identified. Chao1 (83.75–478.96) and Shannon (1.24–6.15) indices showed near-significant differences (Kruskal–Wallis, Chao1: *p* = 0.097; Shannon: *p* = 0.051), suggesting interactive effects of season, region, and root type. Rainy-season CR samples had higher diversity (Chao1: 285.3 ± 124.7; Shannon: 3.8 ± 1.4) than dry-season CR samples (Chao1: 172.4 ± 63.5; Shannon: 2.9 ± 1.1). Regional specificity was evident: e.g., DSYX_CR samples had higher Chao1 than NR, while RSYJ_CR samples had lower Shannon than NR. RS samples from Yingjiang and Lancang showed higher diversity, likely due to increased nutrient availability with precipitation, whereas extreme dry-season samples had reduced diversity, suggesting drought-driven community simplification.

**Figure 4:**
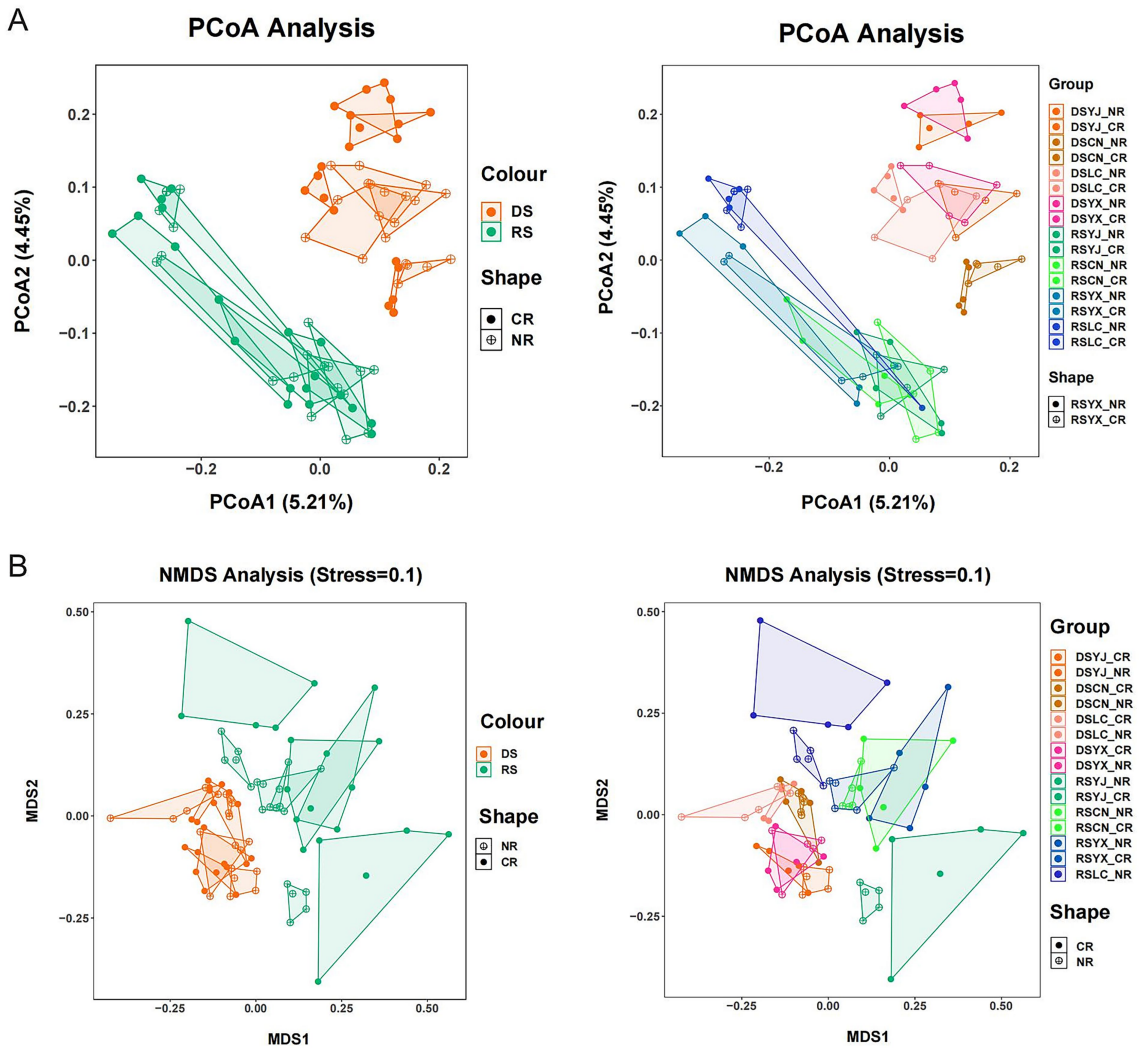
Analysis of beta diversity of macadamia nut rhizosphere fungal communities. PCoA **(A)** and NMDS **(B)** methods were used to characterize the beta diversity of macadamia nut rhizosphere fungal communities in different environments, respectively. DS, dry season; RS, rainy season; CR, cluster roots; NR, normal roots; CN, Changning; YJ, Yingjiang; LC, Lancang; YX, Yunxian.

PERMANOVA indicated season (*p* = 0.001, 3% variance), location (*p* = 0.001, 12% variance), and their interaction (*p* = 0.001, 5% variance) significantly shaped fungal community structure, while root type and its interactions did not (*p* > 0.05; Table 2). PCoA and NMDS (Stress = 0.10) confirmed these patterns, with clear separation of DS vs. RS samples and distinct clustering by location, while root-type effects were minimal (Figure 4). These results highlight season and geography as primary drivers of β-diversity.

**Table 2:** Nonparametric multifactorial ANOVA on the effect of different seasons, locations and root types on the rhizosphere soil fungal community of macadamia nuts.

Factors	*Df*	*R* ^2^	*F* value	*p*
Season	1	0.03	2.91	0.001
Locations	3	0.12	3.69	0.001
Season × Locations	3	0.05	1.68	0.001
Root type	1	0.02	1.99	0.072
Root type × Locations	3	0.03	1.06	0.303
Season × Root type	1	0.01	0.98	0.487
Season × Locations × Root type	3	0.03	0.81	0.946

*Df*, degree of freedom; *R*^2^, goodness of fit; *F*, *F* value; *p*, significance.

### 3.3 Community structure of rhizosphere fungi

**Figure 5:**
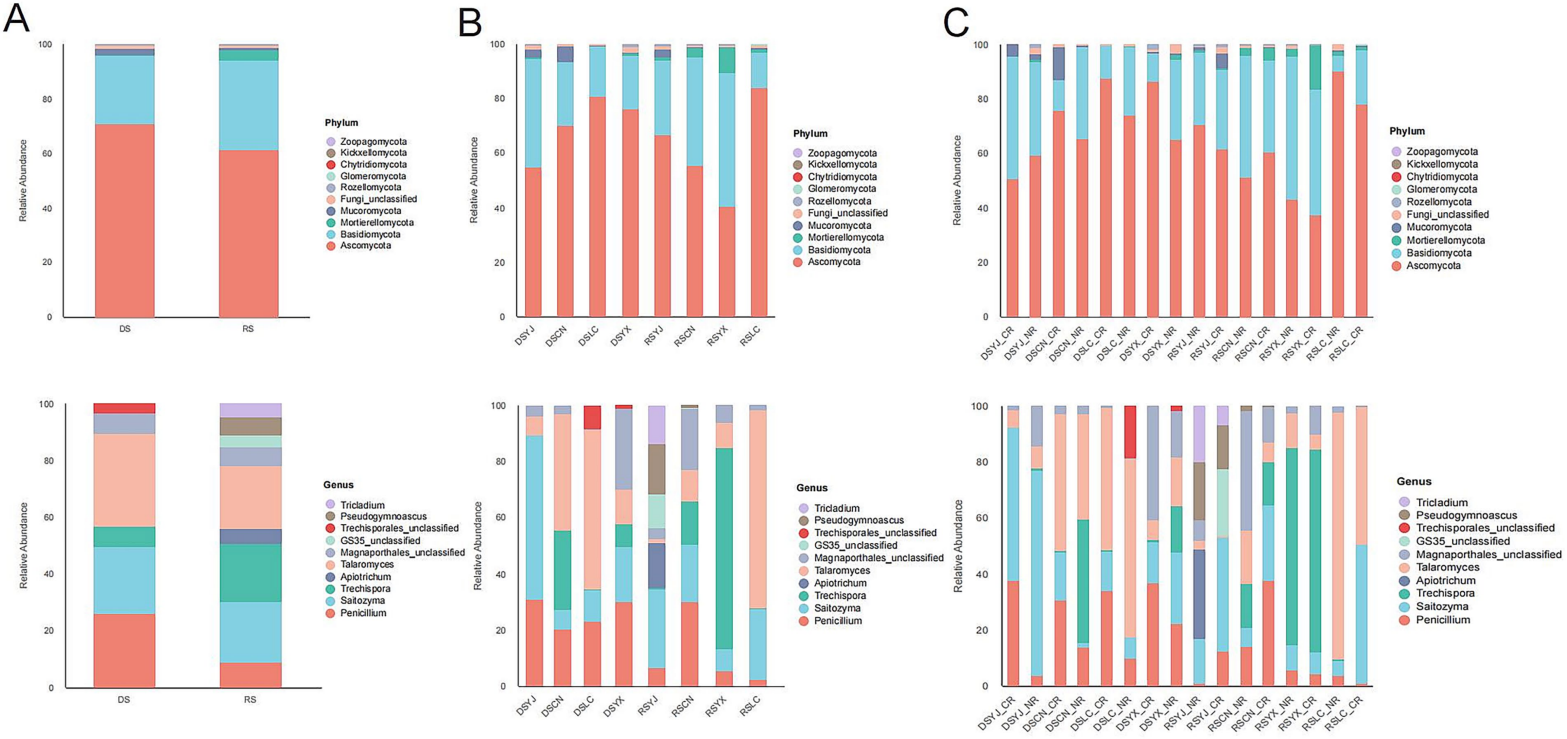
Characterization of macadamia rhizosphere fungal communities in different groups at the phylum and genus levels of abundance. **(A)** Distribution of species at the phylum and genus levels in different seasons; **(B)** Differences in community structure at the phylum and genus levels are presented according to seasonal and regional groupings; **(C)** Combined seasonal, regional, and root type classifications are shown to characterize the composition at the phylum and genus levels, respectively. DS, dry season; RS, rainy season; CR, cluster roots; NR, normal roots; CN, Changning; YJ, Yingjiang; LC, Lancang; YX, Yunxian.

Taxonomic analysis (Figure 5) showed Ascomycota dominated all groups (55–65%) at the phylum level, with Basidiomycota (20–30%) and Mortierellomycota (5–10%) as secondary phyla. Seasonal and spatial variations were more pronounced at lower taxonomic levels: e.g., RS samples had higher abundances of Basidiomycota genera (*Agaricus*, *Hebeloma*) and Ascomycota genera (*Fusarium*, *Acremonium*), while DS samples were enriched in *Mortierella* and *Penicillium* (Ascomycota). Yunxian (YX) had higher Basidiomycota, and Lancang (LC) had more Mortierellomycota. At the genus level, cluster roots (CR) were enriched in symbiotic taxa (e.g., *Glomus*-related), while normal roots (NR) had more saprophytes (e.g., *Trichoderma*). These patterns indicate stability at the phylum level but environmental responsiveness at finer scales.

### 3.4 Key fungal taxa associated with seasonal and regional factors

**Figure 6:**
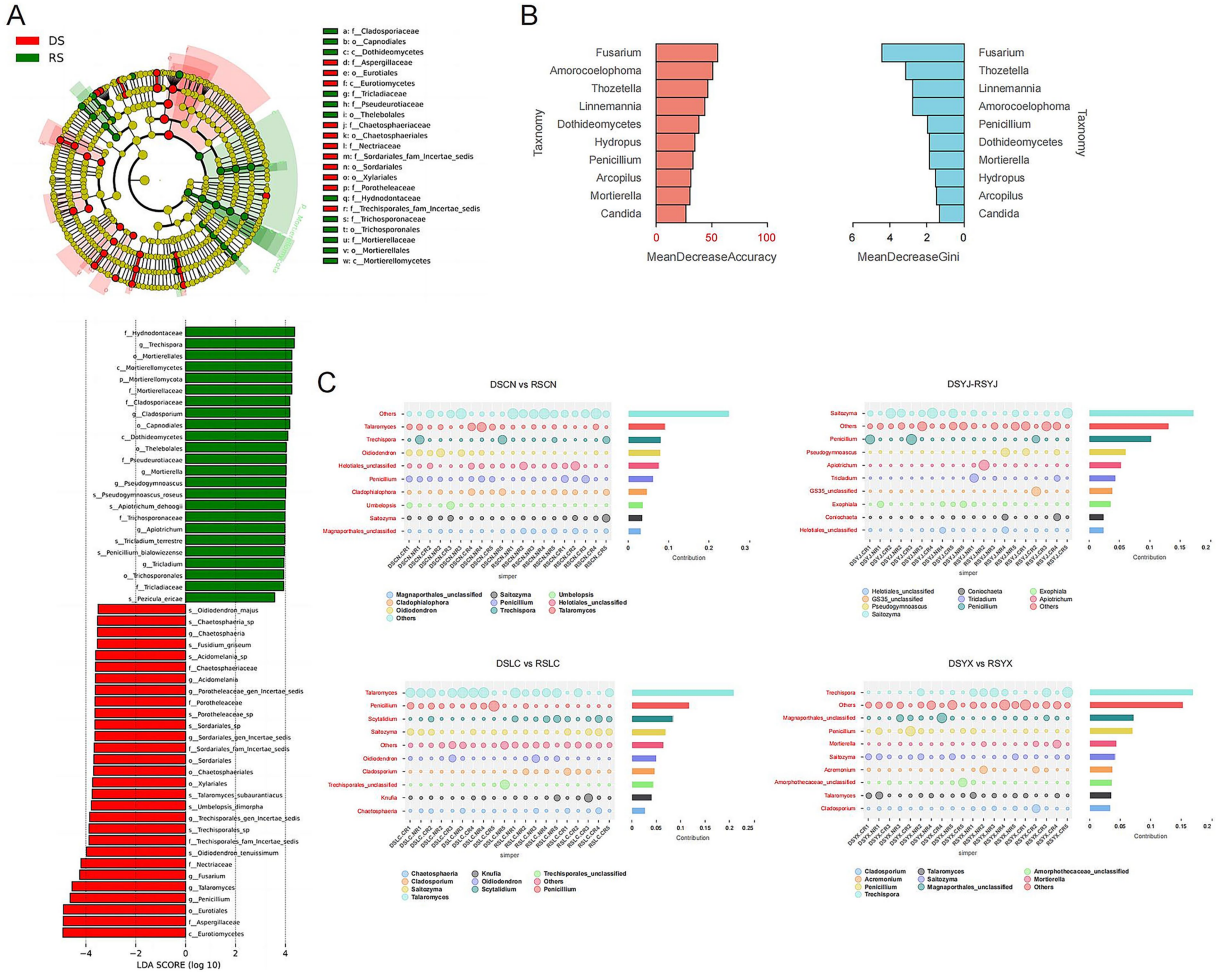
Analysis of differences in rhizosphere fungi of macadamia nuts. **(A)** LEfSe analysis of rhizosphere fungi in the DS and RS. **(B)** Random forest analysis to assess the contribution of key fungal taxa to seasonal variation factors. **(C)** Fungal taxonomic units with significant differences between seasons and root types in each region visualized by effect values based on the Simper analysis method. DS, dry season; RS, rainy season; CR, cluster roots; NR, normal roots; CN, Changning; YJ, Yingjiang; LC, Lancang; YX, Yunxian.

Differential analyses identified key taxa associated with season and location (Figure 6). LEfSe (LDA > 3.5, *p* < 0.05) showed dry-season enrichment of *Mortierella* and *Solicoccozyma*, and rainy-season enrichment of *Vishniacozyma*, *Filobasidium*, and *Fusarium* (pathogenic; Figure 6A). Random Forest confirmed *Penicillium*, *Hypocrea*, *Trichoderma*, and *Fusarium* as top seasonal predictors (Figure 6B). SIMPER analysis revealed region-specific drivers: e.g., *Mortierella* and *Penicillium* drove seasonal differences in Changning; *Fusarium* and *Acremonium* distinguished CR vs. NR; *Metarhizium* and *Aspergillus* increased in rainy seasons in Lancang and Yunxian (Figure 6C).

### 3.5 Co–occurrence network analysis of rhizosphere fungi in different environments

**Figure 7:**
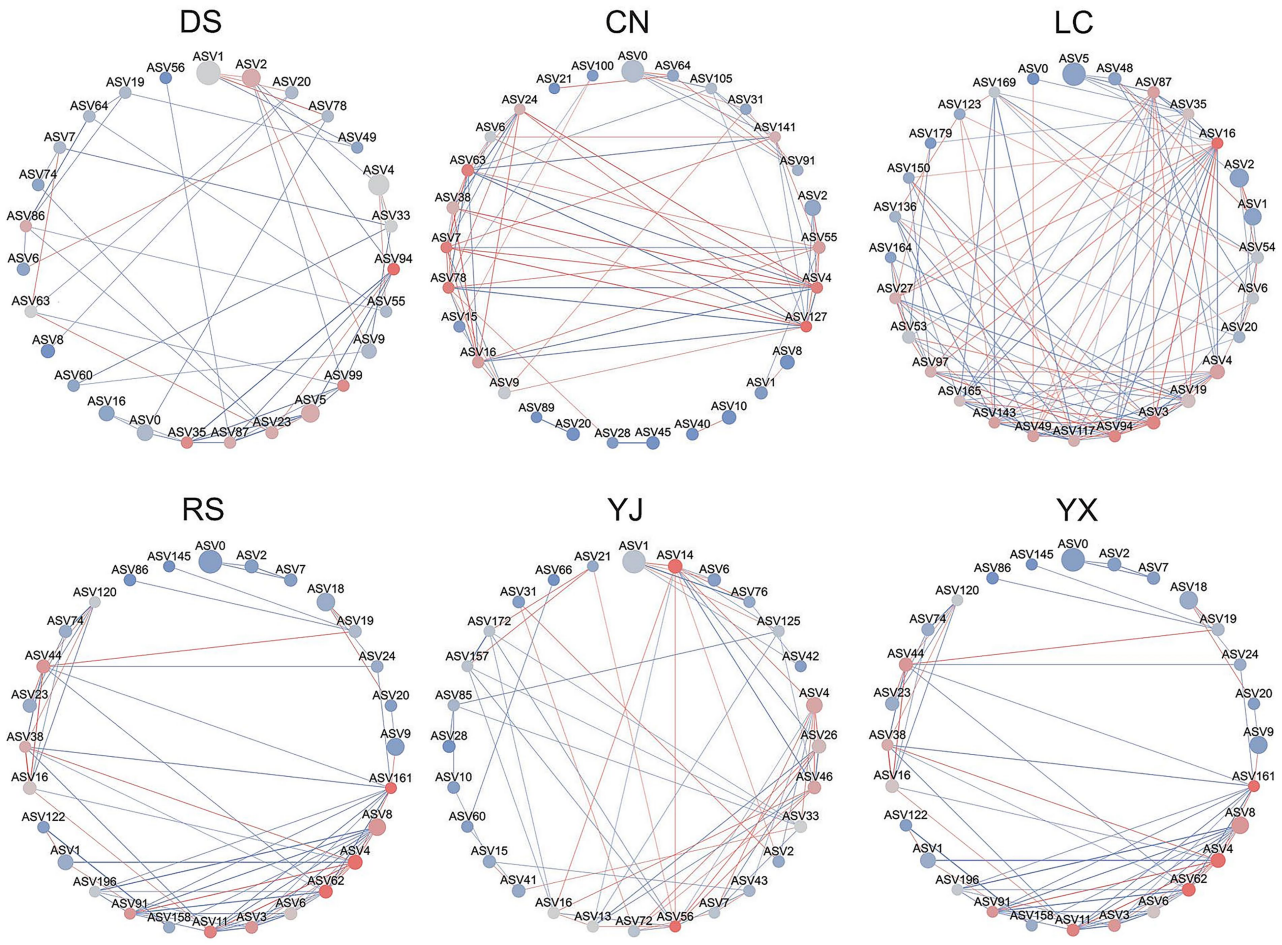
Network analysis of co–occurrence of rhizosphere fungi under different environmental conditions. In each network diagram, nodes represent ASVs, and node colors in red indicate high abundance, gray indicates medium abundance, and blue indicates low abundance. Node size indicates the degree value of the interactions, and larger nodes indicate larger degree values. The connecting lines between the nodes indicate the interactions between the nodes, red color indicates positive correlation and blue color indicates negative correlation. DS, dry season; RS, rainy season; CR, cluster roots; NR, normal roots; CN, Changning; YJ, Yingjiang; LC, Lancang; YX, Yunxian.

Co-occurrence network analysis revealed environment-specific interaction patterns (Figure 7; Table 3). Dry-season networks were dominated by Ascomycota (*Talaromyces*, *Penicillium*) with high degree and betweenness centrality, while rainy-season networks featured *Cladosporium* (Ascomycota) and Mortierellaceae (Mortierellomycota) as keystone taxa. Regionally, keystone species varied: e.g., *Phialomyces* in Changning, *Cladosporium* and *Talaromyces* in Lancang, and *Penicillium* with *Acremonium* in Yunxian. These patterns highlight environmental shaping of network structure.

**Table 3:** The potential keystone species (ASVs) in the rhizosphere fungal co–occurrence network of macadamia nuts in different environments.

Environments	ASV ID	Sum_Abundance	Degree	Betweenness centrality	Phylum	Family/Genus
DS	ASV94	22,404	7	104.53	Ascomycota	*Talaromyces*
	ASV99	20,350	6	67.33	Ascomycota	*Penicillium*
	ASV35	19,350	6	72.73	Ascomycota	*Talaromyces*
	ASV2	164,824	5	110.47	Ascomycota	*Talaromyces*
	ASV5	160,110	5	24.13	Ascomycota	*Talaromyces*
RS	ASV24	31,250	10	113.64	Mortierellomycota	Mortierellaceae
	ASV3	67,111	9	209.15	Ascomycota	*Cladosporium*
	ASV8	93,563	8	70.94	Basidiomycota	*Trechispora*
	ASV51	33,218	8	53.11	Ascomycota	*Talaromyces*
	ASV7	26,025	7	60.88	Ascomycota	*Acidomelania*
CN	ASV127	14,693	12	70.16	Ascomycota	*Phialomyces*
	ASV4	19,560	11	23.71	Ascomycota	*Penicillium*
	ASV78	21,719	11	138.65	Ascomycota	*Oidiodendron*
	ASV7	16,643	11	29.26	Ascomycota	*Acidomelania*
	ASV63	32,367	11	39.74	Ascomycota	*Oidiodendron*
LC	ASV16	12,912	16	94.61	Ascomycota	*Fusarium*
	ASV3	43,307	14	78.44	Ascomycota	*Cladosporium*
	ASV94	20,779	14	37.27	Ascomycota	*Talaromyces*
	ASV87	24,014	12	22.75	Ascomycota	*Penicillium*
	ASV4	65,799	12	41.44	Ascomycota	*Penicillium*
YJ	ASV14	67,409	11	179.04	Ascomycota	*Pseudogymnoascus*
	ASV56	9,868	11	108.60	Ascomycota	*Chaetosphaeria*
	ASV4	111,349	8	106.19	Ascomycota	*Penicillium*
	ASV46	47,264	8	26.20	Ascomycota	*Tricladium*
	ASV26	67,938	7	16.75	Basidiomycota	*Apiotrichum*
YX	ASV161	9,927	10	138.80	Basidiomycota	*Trechispora*
	ASV4	49,833	10	99.22	Ascomycota	*Penicillium*
	ASV62	34,958	10	73.20	Ascomycota	*Acremonium*
	ASV11	22,052	9	23.74	Ascomycota	*Cladosporium*
	ASV8	84,940	8	48.64	Basidiomycota	*Trechispora*

### 3.6 Functional predictive analysis based on FUNGuild

**Figure 8:**
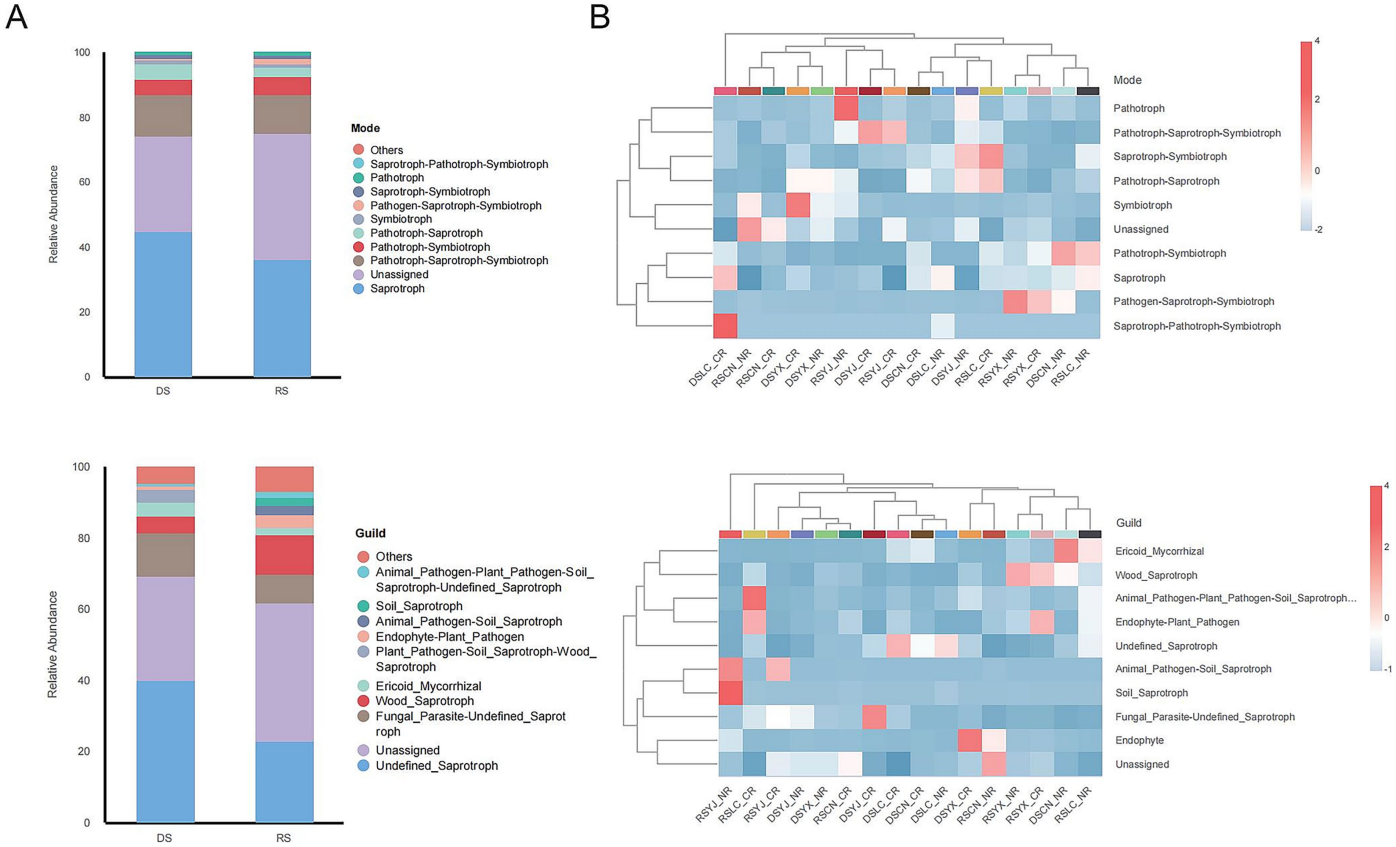
Predictive analysis of rhizosphere fungal function based on the FUNGuild database. **(A)** Bar stacked plots of functional patterns and relative abundance of functional groups of fungi in the DS and RS. **(B)** Clustered heat maps of functional patterns and functional group distribution of rhizosphere fungi in different groups that combined season, region, and root type. DS, dry season; RS, rainy season; CR, cluster roots; NR, normal roots; CN, Changning; YJ, Yingjiang; LC, Lancang; YX, Yunxian.

FUNGuild predictions (Figure 8) showed saprotrophic fungi dominated (50–55%), with a 10% higher proportion in rainy vs. dry seasons. Guild analysis revealed Unassigned and Soil_Saprotroph as primary groups, with seasonal fluctuations in functional groups like Animal_Pathogen–Plant_Pathogen_Soil_Saprotroph. Functional patterns (e.g., Pathotroph–Saprotroph–Symbiotroph) and guilds (e.g., Endo_Mycorrhizal, Wood_Saprotroph) varied with region, season, and root type: e.g., higher Animal_Pathogen–Plant_Pathogen_Soil_Saprotroph in DSCN_CR, and prominent Soil_Saprotroph in RSYX_NR. These patterns indicate environmental factors drive functional adaptation of rhizosphere fungi.

## 4 Discussion

### 4.1 The formation of rhizosphere fungal community structure is primarily driven by seasonal and regional differences

Our results strongly support the hypothesis that season and geographic location are primary drivers of rhizosphere fungal community structure. PERMANOVA analysis revealed significant effects of season (*p* = 0.001, 3% variance explained), location (*p* = 0.001, 12% variance explained), and their interaction (*p* = 0.001, 5% variance explained) on community structure, whereas root type alone had no significant effect (*p* = 0.072; Table 2). These findings align with the conclusion that climatic variables dominate fungal community composition (Bui et al., 2020) but extend it by identifying specific mechanisms through our data:

Seasonal effects: Alpha diversity indices (Chao1: 285.3 ± 124.7; Shannon: 3.8 ± 1.4) were significantly higher in rainy-season CR samples compared to dry-season CR samples (Chao1: 172.4 ± 63.5; Shannon: 2.9 ± 1.1; Figure 3), consistent with increased soil moisture and root exudates (e.g., sugars, amino acids) in the rainy season stimulating fungal proliferation (Jiang et al., 2023). Taxonomic analysis further showed that rainy-season samples were enriched in saprotrophic taxa such as *Fusarium* and *Acremonium* (Figures 5, 6), validating our prediction that high humidity accelerates organic matter decomposition and supports saprotroph growth. In contrast, dry-season dominance of *Mortierella* (Figure 6A) likely reflects adaptation to drought via accumulation of osmolytes like proline (Kaushal, 2019).

Regional effects: Geographic variation in dominant phyla—e.g., higher Mortierellomycota in Lancang (LC; low elevation, 992 m; high rainfall, 1,429 mm) and higher Basidiomycota in Yunxian (YX; high elevation, 1,278 m; Figure 5)—directly demonstrates environmental filtering of fungal taxa. SIMPER analysis identified region-specific drivers, such as increased *Metarhizium* in rainy-season Lancang (Figure 6C), reinforcing the role of local microclimates in shaping communities (Tao et al., 2020).

### 4.2 Cluster roots have an enrichment effect on fungi under specific conditions

Our results partially support the hypothesis that cluster roots selectively enrich symbiotic fungi, with enrichment dependent on environmental conditions. While root type alone had no significant effect (PERMANOVA, *p* = 0.072), cluster roots (CR) showed 1.8- and 2.3-fold higher relative abundances of *Glomus* and *Trichoderma*, respectively, compared to normal roots (NR; Figure 5), consistent with the prediction that cluster roots recruit beneficial taxa via exudates (Zhao et al., 2021).

Notably, this enrichment was context-dependent: Chao1 indices were significantly higher in dry-season CR samples from Yunxian (DSYX_CR) compared to DSYX_NR (Figure 3A), but no such difference was observed in rainy-season Lancang (RSLC), likely due to dilution of root exudates by heavy rainfall (Figure 2B). This suggests cluster root effects are amplified in seasons/regions where root exudates remain concentrated (e.g., dry-season Yunxian), supporting our hypothesis that environmental conditions modulate root-fungus interactions.

### 4.3 The structure of key fungal taxa and ecological functions is strongly driven by environmental factors

Key taxa (*Fusarium*, *Mortierella*, *Trichoderma*, *Penicillium*) identified by LEfSe and Random Forest act as environmental indicators. Rainy-season enrichment of *Fusarium* (pathogenic) likely reflects favorable growth conditions under high humidity (Popovski and Celar, 2013), while dry-season dominance of *Mortierella* (drought-tolerant saprotroph) indicates adaptation to resource-limited conditions (Caravaca et al., 2024). FUNGuild analyses supported the hypothesis that functional guild composition shifts with seasonal and regional changes in community structure. Saprotrophs dominated in both seasons but were 10% more abundant in the rainy season (50–55% vs. dry season; Figure 8), consistent with increased organic matter decomposition under high moisture (Tanunchai et al., 2023). Conversely, dry-season samples showed higher proportions of multi-trophic guilds (e.g., Animal_Pathogen–Plant_Pathogen–Soil_Saprotroph; Figure 8B), reflecting fungal adaptation to resource scarcity via facultative parasitism.

Key taxa identified by LEfSe–e.g., rainy-season enrichment of Fusarium (pathogen) and Trichoderma (biocontrol agent; Figure 6A)–further validated functional differentiation under high humidity. Dry season dominance of drought–tolerant *Mortierella* (saprotroph; Figure 6A) aligned with our prediction of stress–driven functional specialization. Additionally, 35% of edges in rainy season co–occurrence networks were negative (Figure 7), indicating intensified resource competition among saprotrophs, directly linking community structure to functional dynamics.

### 4.4 Co–occurrence network analysis reveals key taxa for rhizosphere fungal interactions

Co-occurrence networks revealed environmental shifts in fungal interactions. Dry-season networks were dominated by Ascomycota (*Talaromyces*, *Penicillium*) with high connectivity, forming mutualistic modules (e.g., with *Trichoderma*) likely involved in cellulose decomposition and nutrient cycling (He et al., 2017, 2025; Samson et al., 2011). Rainy-season networks shifted to *Cladosporium* and Mortierellaceae, with 35% negative interactions indicating heightened resource competition—likely due to rapid organic matter decomposition limiting carbon availability. Regional variations in keystone species (e.g., *Phialomyces* in Changning, *Cladosporium* in Lancang) highlight geographic shaping of network structure (Liu et al., 2024; Zhang et al., 2017, 2018). *Penicillium*, a prevalent hub across networks, may act as a ‘resident’ core taxon, warranting functional validation.

## 5 Conclusion

This study provides a comprehensive analysis of the seasonal and regional dynamics of rhizosphere fungal communities in *Macadamia integrifolia*, offering new insights into the ecological roles of fungi in tropical crops. The results underscore the importance of environmental factors–particularly seasonality and geographic location–in shaping fungal community structure and functional composition. Notably, the higher diversity observed in the rainy season suggests a strong link between environmental moisture and fungal proliferation, while the dominance of saprotrophic fungi highlights the role of these organisms in nutrient cycling. Cluster roots were found to selectively enrich symbiotic fungi, such as Glomus and Trichoderma, thereby potentially enhancing plant acclimatization under specific environmental conditions. These findings also reveal the adaptive strategies of fungal communities in response to seasonal and regional variations. Overall, this research contributes to the understanding of rhizosphere fungal dynamics and lays a foundation for the development of microbiome-based strategies to improve macadamia cultivation, emphasizing the role of fungal biodiversity in ecosystem health and plant productivity.

## Data Availability

The datasets presented in this study can be found in online repositories. The names of the repository/repositories and accession number(s) can be found in the article/Supplementary material.
